# RETRACTION: Assessing Mailuoning Injection in Wound Healing and Thrombophlebitis Management: A Rat Model Study

**DOI:** 10.1111/iwj.70476

**Published:** 2025-04-02

## Abstract

**RETRACTION:** XiaoJ. , XieY. , LiuJ. , LiuT. , YeR. , DuanX. , LeZ. , DengN. , and DuanQ. , “Assessing Mailuoning Injection in Wound Healing and Thrombophlebitis Management: A Rat Model Study,” International Wound Journal 21, no. 4 (2024): e14527, 10.1111/iwj.14527.38095110 PMC10961041

The above article, published online on 14 December 2023, in Wiley Online Library (http://onlinelibrary.wiley.com/), has been retracted by agreement between the journal Editor in Chief, Professor Keith Harding; and John Wiley & Sons Ltd. Following an investigation by the publisher, all parties have concluded that this article was accepted solely on the basis of a compromised peer review process. In addition, the authors provided incomplete ethical approval information for the study. The editors have therefore decided to retract the article. The authors did not respond to our notice regarding the retraction.

**RETRACTION:** J. Xiao , Y. Xie , J. Liu , T. Liu , R. Ye , X. Duan , Z. Le , N. Deng , and Q. Duan , “Assessing Mailuoning Injection in Wound Healing and Thrombophlebitis Management: A Rat Model Study,” International Wound Journal 21, no. 4 (2024): e14527, 10.1111/iwj.14527.38095110 PMC10961041

The above article, published online on 14 December 2023, in Wiley Online Library (http://onlinelibrary.wiley.com/), has been retracted by agreement between the journal Editor in Chief, Professor Keith Harding; and John Wiley & Sons Ltd. Following an investigation by the publisher, all parties have concluded that this article was accepted solely on the basis of a compromised peer review process. In addition, the authors provided incomplete ethical approval information for the study. The editors have therefore decided to retract the article. The authors did not respond to our notice regarding the retraction.

